# Monitoring of gadolinium uptake within the vessel wall during magnetic resonance (MR) guided angioplasty of the peripheral arteries with a paclitaxel/gadolinium coated balloon: an experimental study at 3 T

**DOI:** 10.1186/1532-429X-13-S1-O58

**Published:** 2011-02-02

**Authors:** Mirja Neizel, Michael Perkuhn, Jan Balzer, Alexandra Buhl, Alexander Rübben, Norbert Weiss, Malte Kelm, Gabriele A Krombach

**Affiliations:** 1 University Hospital Duesseldorf, Düsseldorf, Germany; 2 University Hospital Aachen, Aachen, Germany; 3 University Hospital Giessen, Giessen, Germany; 4 Aachen Resonance, Aachen, Germany

## Introduction

Stenotic peripheral vessels often re-occlude after treatment with an angioplasty balloon catheter. Recently, angioplasty catheters with paclitaxel applied to the balloon have been developed. However, distribution of the substance within the vessel wall cannot be monitored under x-ray guidance

## Purpose

The aim of this study was to demonstrate the feasibility of monitoring the delivery of paclitaxel/gadolinium to the vessel wall during MR guided angioplasty of the iliac arteries using a paclitaxel/gadolinium coated balloon catheter.

## Methods

6 pigs (47±2 kg) were investigated. All experiments were performed using a 3 Tesla MRI system using a 6 channel coil. Iliaca-Interventions were performed using a preclinical paclitaxel/gadolinium coated balloon catheter and were monitored using a SSFP real-time imaging sequence. The feasibility of monitoring the delivery of paclitaxel/gadolinium to the vessel wall was assessed in 4 animals. In two animals stenosis were surgically induced to the iliac arteries (severity of stenosis 76-99% confirmed by MR angiography). Delivery of paclitaxel/gadolinium to the vessel wall was monitored using a 3D T1-weighted gradient echo (GE) sequence for delineation of the vessel wall. Normalized signal intensity of the vessel wall was measured before and after the angioplasty in all animals.

## Results

**Figure 1:**
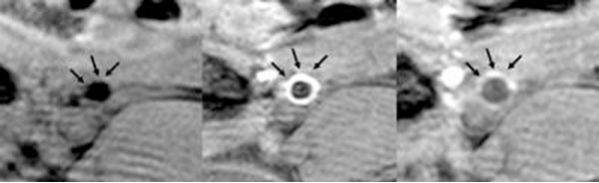
On the left the vessel wall before angioplasty shows no enhancement. In the middle the T1-weighted GE image directly after angioplasty illustrates homogenous enhancement of the vessel wall suggesting uptake of the paclitaxel/gadolinium solution. 30 minutes after angioplasty the enhancement of the vessel wall has already decreased (right image)

Angioplasty with a paclitaxel/gadolinium coated balloon was successfully performed in all animals without stenosis (n=8). In animals with created stenosis, MR-angiography demonstrated successful dilatation in all cases (n=4). 3 stenosis were completely removed after angioplasty and one stenosis was mild. The normalized signal intensity of the vessel wall significantly increased after the intervention on the T1 weighted GE images in all animals (p<0.01). Figure 1.

## Conclusions

Monitoring the delivery of paclitaxel/gadolinium within the vessel wall during MR guided coated balloon angioplasty of the iliac arteries is feasible. This may help to confirm the success of uptake of paclitaxel to the vessel wall, which is known to reduce the restenosis rate after angioplasty.

